# A comparative study of transperineal software-assisted magnetic resonance/ultrasound fusion biopsy and transrectal cognitive fusion biopsy of the prostate

**DOI:** 10.1186/s12894-022-01011-w

**Published:** 2022-04-29

**Authors:** Po-Fan Hsieh, Tian-You Chang, Wei-Ching Lin, Han Chang, Chao-Hsiang Chang, Chi-Ping Huang, Chi-Rei Yang, Wen-Chi Chen, Yi-Huei Chang, Yu-De Wang, Wen-Chin Huang, Hsi-Chin Wu

**Affiliations:** 1 Department of Urology, China Medical University Hospital, No. 2, Yu-Der Rd, Taichung, 40447 Taiwan; 2 School of Medicine, China Medical University, Taichung, 40402 Taiwan; 3 Graduate Institute of Biomedical Sciences, School of Medicine, China Medical University, Taichung, 40402 Taiwan; 4 Department of Radiology, China Medical University Hospital, Taichung, 40447 Taiwan; 5 Department of Pathology, China Medical University Hospital, Taichung, 40447 Taiwan; 6 Department of Urology, China Medical University Beigang Hospital, Beigang, Yunlin, 651012 Taiwan

**Keywords:** Clinically significant prostate cancer, MR/US fusion biopsy, Prostate biopsy, Transperineal, Transrectal

## Abstract

**Background:**

The advantages and disadvantages of transperineal and transrectal biopsies remain controversial in the era of prostate targeted biopsy. In this study, we compared the cancer detection and complication rates of transperineal magnetic resonance/ultrasound (MR/US) fusion biopsy and transrectal cognitive fusion biopsy of the prostate.

**Methods:**

This was a comparative study of two prospectively collected cohorts. Men with clinically suspected prostate cancer and prostate imaging reporting and data system (PI-RADS) score ≥ 3 lesions on multi-parametric magnetic resonance imaging (mpMRI) were enrolled. They underwent either transperineal software fusion biopsy or transrectal cognitive fusion biopsy and systematic biopsy. The detection rates of any prostate cancer and clinically significant prostate cancer (csPC, defined as Gleason score ≥ 3 + 4) and the complication rates between both groups were analysed.

**Results:**

Ninety-two and 85 patients underwent transperineal software fusion and transrectal cognitive fusion biopsies, respectively. The detection rate for any prostate cancer was similar between both groups (60.8% vs. 56.4%, *p* = 0.659). In terms of csPC detection, transperineal fusion biopsy outperformed transrectal fusion biopsy (52.2% vs. 36.5%, *p* = 0.036). In multivariate regression analysis, age, PI-RADS score > 3, and transperineal route were significant predictors of csPC. Meanwhile, transperineal biopsy resulted in a higher rate of urinary retention than transrectal biopsy (18.5% vs. 4.7%, *p* = 0.009). No serious infectious complications were noted, although a patient developed sepsis after transrectal biopsy.

**Conclusions:**

Transperineal software fusion biopsy provided a higher csPC detection rate than transrectal cognitive fusion biopsy and carried minimal risk for infectious complications in patients with MRI-visible prostate lesions.

**Supplementary Information:**

The online version contains supplementary material available at 10.1186/s12894-022-01011-w.

## Introduction

Transrectal ultrasound (TRUS)-guided systematic biopsy (SB) of the prostate has long been the standard for diagnosing prostate cancer [1]. However, SB is usually random in nature, and undersampling of the prostate missed approximately 20–30% of clinically significant prostate cancer (csPC) [2, 3]. Besides, TRUS-guided biopsy often leads to the overdiagnosis of clinically insignificant prostate cancer (ciPC). Due to the great improvement in multi-parametric magnetic resonance imaging (mpMRI), targeted biopsy (TB) has emerged as a promising imaging tool in the diagnosis of csPC [4]. Currently, both European Association of Urology (EAU) and American Urological Association (AUA) guidelines recommend mpMRI before initial or repeated prostate biopsy [5, 6]. MR/US fusion biopsy of the prostate can be performed using software or cognitive fusion. However, current literature shows no significant difference in cancer detection rates between software fusion and cognitive fusion biopsies [7, 8].

During biopsy, prostatic tissue can be sampled from the transrectal or transperineal route. Transrectal biopsy is readily accessible in an office setting, whereas transperineal biopsy causes more perineal pain and usually requires general or spinal anaesthesia. Regarding diagnostic accuracy, it has been reported that cancer detection rates were comparable between transrectal and transperineal biopsies without pre-biopsy mpMRI [9, 10]. In the era of MRI-TB, only a few studies have compared cancer detection rates between transrectal and transperineal biopsies; however, the results obtained were conflicting [11–14]. This study aimed to compare cancer detection and complication rates between transrectal cognitive fusion and transperineal MR/US software fusion biopsies.

## Materials and methods

### Study population

After approval by the Research Ethics Committee of our institute (protocol numbers: CMUH105-REC1-123 and CMUH109-REC1-045), we prospectively collected two cohorts of MRI-TB including transrectal cognitive fusion biopsy and transperineal MR/US software fusion biopsy. The inclusion criteria were men who were above 40 years and had abnormal findings on digital rectal examination (DRE) or serum prostate-specific antigen (PSA) level ≥ 4 ng/mL. We enrolled men with positive findings (Prostate Imaging-Reporting and Data System [PI-RADS] ≥ 3) on pre-biopsy mpMRI [15]. Men with a history of prostate cancer, bacterial prostatitis within 3 months, use of 5-alpha reductase inhibitors, or inability to provide informed consent were excluded. We recorded and reported prostate biopsy results following the Standards of Reporting for MRI-Targeted Biopsy Studies (START) guidelines (Additional file 1: Supplementary table) [16]. All methods were performed in accordance with EAU guidelines. Informed consent was obtained from all the patients.

### MRI protocol

All mpMRI scans were performed using a 3-T scanner (Signa HDxt; GE Healthcare, Milwaukee, WI, USA) with a four-channel high-definition (HD) cardiac array coil. The scanning protocol included T2 weighted imaging (T2WI), diffusion-weighted imaging (DWI), apparent diffusion coefficient mapping, and dynamic contrast enhancement. The slice thickness was 3 mm. The axial, sagittal, and coronal planes of T2WI were acquired. DWI was acquired with b-values of 0 and 1000 s/mm^2^. The temporal resolution of the DCE was 7–10 s without a post-processing model. The acquisition and reporting of mpMRI corresponded with PI-RADS v2 [15]. An experienced uroradiologist (W.C.L.) with 12 years of prostate MRI experience interpreted these images without being blinded to clinical information and marked the targeted lesions on T2WI. These targeted lesions were used for cognitive fusion or software fusion biopsies.

### Biopsy protocol

Transrectal cognitive fusion biopsy was performed from 2016 to 2018. The patients underwent intravenous general anaesthesia and were placed in a lateral decubitus position. At least two biopsy cores were obtained from each target, and at least 12 cores were systematically collected. We introduced the MR/US fusion platform BioJet™ (D&K Technologies, Barum, Germany) in 2019 and performed transperineal MR/US software fusion biopsy exclusively. The patients underwent general or spinal anaesthesia and were placed in a lithotomy position. At least three cores were taken from each target, and SB was performed following the Ginsburg protocol with at least 12 cores [17]. All biopsies were performed by a single urologist (P.F.H.), who had performed cognitive fusion biopsy in more than 150 cases before 2016. All biopsy specimens were interpreted by an experienced uropathologist (H.C.), and prostate cancer was graded according to the 2014 International Society of Urological Pathology Consensus Conference guidelines [18]. CsPC was defined as Gleason score ≥ 3 + 4 or Gleason grade group ≥ 2 [19]. CiPC was defined as Gleason score ≦3 + 3 or Gleason grade group 1.

### Outcome measures and statistical analysis

Continuous variables are reported as medians (interquartile range [IQR]), and categorical variables are reported as proportions. Patient characteristics were compared between the transrectal cognitive fusion biopsy group and the transperineal fusion biopsy group using the chi-square or Mann–Whitney U test as appropriate. Cancer detection rates between the two cohorts were then compared. Subgroup analysis was performed according to the PI-RADS score and target lesion location. A logistic regression analysis was performed to identify the predictors of csPC. Finally, the complication rates between these two cohorts were compared according to the Clavien-Dindo classification. Statistical analyses were conducted using SPSS version 22 (IBM Corp., Armonk, NY, USA), and a two-sided test with an alpha of 5% was considered statistically significant.

## Results

**Fig. 1:**
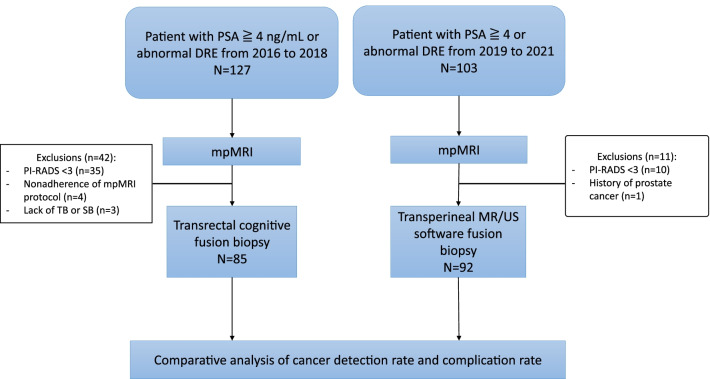
Flowchart of the study design and participants. DRE, digital rectal examination; mpMRI, multi-parametric magnetic resonance imaging; PI-RADS, Prostate Imaging-Reporting and Data System; PSA, prostate-specific antigen; SB, systematic biopsy; TB, targeted biopsy; US, ultrasonography

A total of 177 men were consecutively enrolled in this study, including 85 in the transrectal cognitive fusion biopsy group and 92 in the transperineal software fusion biopsy group (Fig. 1). The patient characteristics are shown in Table 1. The median age, PSA level, prostate volume, and distribution of PI-RADS scores were compared between both groups. The median number of biopsy cores was higher in the transperineal biopsy group than in the transrectal biopsy group (26 vs. 20, *p* < 0.001).

**Table 1:** Patient characteristics

	Transperineal biopsy (N = 92)	Transrectal biopsy (N = 85)	*p*
Total number of targets	104	92	
Age	66 (61–72)	67 (62–73)	0.61
DRE, n (%)			0.312
Negative	65 (70.6%)	62 (72.9%)	
Positive	27 (29.3%)	23 (27.0%)	
Previous biopsy, n (%)			0.844
Biopsy naïve	64 (69.5%)	57 (67.0%)	
Repeat biopsy	28 (30.4%)	28 (32.9%)	
PSA, ng/mL	7.5 (5.30–13.28)	8.04 (5.76–12.12)	0.608
Prostate volume, ml	42.34 (29.93–59.08)	43.4 (30.9–54.9)	0.582
PSAD	0.18 (0.11–0.32)	0.18 (0.12–0.27)	0.729
PI-RAS score, n (%)			0.277
3	19 (18.2%)	13 (14.1%)	
4	48 (46.1%)	53 (57.6%)	
5	37 (35.5%)	26 (28.2%)	
Total core number, n	26 (23–28)	20 (16–22)	< 0.001
Targeted biopsy	6 (4–7)	4 (3–4)	< 0.001
Systematic biopsy	19 (17–22)	16 (12–18)	< 0.001
Target lesion size, mm	12 (9–19.25)	10.5 (6.75–16)	0.305
Target lesion location, n (%)			
Anterior	41 (39.4%)	31 (33.6%)	0.406
posterior	63 (60.5%)	61 (66.3%)	

Continuous data were show as median (IQR)

DRE, digital rectal exam; IQR, interquadrant range; PSA, prostate specific antigen; PSAD, prostate specific antigen density; PI-RADS, Prostate Imaging Reporting & Data System

**Fig. 2:**
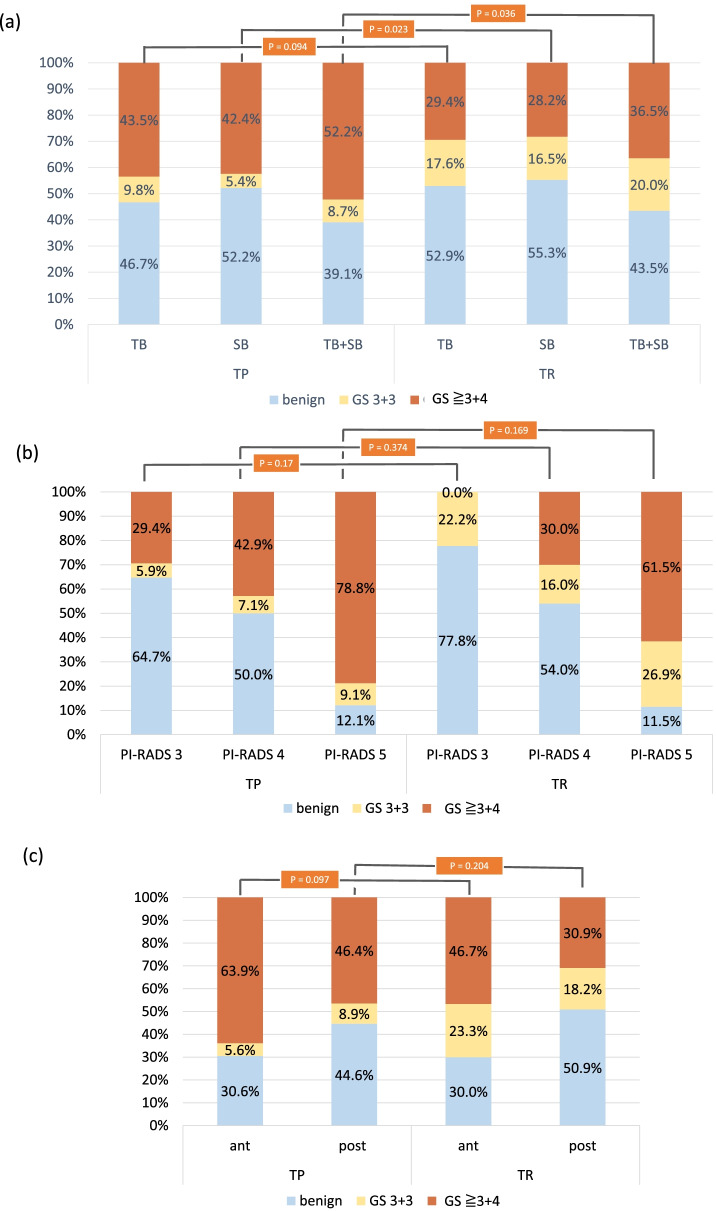
Comparison of the cancer detection rate between transperineal software fusion biopsy and transrectal cognitive biopsy stratified by **a** TB or SB, **b** PI-RADS score, and **c** target lesion location. Each *p* value indicated the comparison of the detection rates for csPC between transperineal biopsy and transrectal biopsy. ant, anterior lesion; csPC, clinically significant prostate cancer; GS, Gleason score; PI-RADS, Prostate Imaging-Reporting and Data System; post, posterior lesion; SB, systematic biopsy; TB, targeted biopsy; TR, transrectal biopsy; TP, transperineal biopsy

The detection rate for any prostate cancer was similar between the two groups (60.9% vs. 56.5%, *p* = 0.659). Regarding csPC detection, the transperineal biopsy group performed better than the transrectal biopsy group (52.2% vs. 36.5%, *p* = 0.036), whereas the transrectal biopsy group detected more ciPC than the transperineal biopsy group (20% vs. 8.7%, *p* = 0.031) (Fig. 2a). The detection rate for csPC by TB was similar between both groups (43.5% vs. 29.4%, *p* = 0.094), and the detection rate for csPC by SB was higher in the transperineal biopsy group (42.2% vs. 28.2%, *p* = 0.023) (Fig. 2a). In contrast, there were no significant differences in the detection rates for csPC between both groups stratified by PI-RADS scores and target lesion locations (Fig. 2b, c).

In univariate regression analysis, age, PI-RADS score > 3, transperineal route, and anterior target lesion location were significant predictors of csPC. In multivariate regression analysis, age, PI-RADS score > 3, and transperineal route remained significant predictors of csPC (Table 2).

**Table 2:** Logistic regression analysis of predictor for csPC

	Univariate			Multivariate		
	OR	95% CI	*p*	OR	95% CI	*p*
Age	1.06	1.02–1.1	0.003	1.05	1.01–1.1	0.023
Prior negative biopsy	0.62	0.32–1.19	0.149			
DRE						
Negative	reference					
Positive	0.58	0.31–1.09	0.089			
PSA	1.04	1.00–1.08	0.055	1.03	0.99–1.06	0.111
PI-RADS						
3	reference					
> 3	5.25	1.72–16.02	0.004	5.99	1.72–20.84	0.005
Number of total cores	1.03	0.98–1.09	0.276			
Biopsy route						
Transperineal	1.99	1.09–3.63	0.026	2.55	1.32–4.95	0.006
Transrectal	Reference					
Target lesion location						
Posterior	Reference					
Anterior	2.02	1.09–3.74	0.026	1.81	0.91–3.57	0.09

CI: confidence interval; csPC: clinically significant prostate cancer; DRE: digital rectal exam; OR: odds ratio; PSA: prostate specific antigen; PI-RADS: Prostate Imaging Reporting & Data System

There were no Clavien-Dindo ≥ 3 complications in either group. Seventeen men in the transperineal biopsy group and four men in the transrectal biopsy group had acute urinary retention (AUR) (18.5% vs. 4.7%, *p* = 0.009). Patients could void well after intermittent catheterisation or Foley catheterisation for 1 day and administration of oral alpha-blockers. One patient (1.2%) in the transrectal biopsy group returned to our emergency department on postoperative day 1 and received intravenously administered antibiotics for sepsis for 7 days. His blood culture yielded *Escherichia coli*. One patient in the transperineal biopsy group and one in the transrectal biopsy group had afebrile urinary tract infection, respectively (Table 3).

**Table 3:** Post-biopsy complications

	Transperineal biopsy	Transrectal biopsy	*p*
	N (%)	N (%)	
AUR	17 (18.5%)	4 (4.7%)	0.009
UTI	1 (1.1%)	1 (1.2%)	1
Sepsis	0 (0.0%)	1 (1.2%)	0.968
Gross hematuria	11 (11.9%)	17 (20%)	0.155
Hemospermia	0	1 (1.2%)	0.968
Pain on biopsy site	6 (6.5%)	3 (3.5%)	0.5
Rectal bleeding	–	3 (3.5%)	
Perineal hematoma	3 (3.2%)	–	

AUR, acute urine retention; UTI, urinary tract infection

## Discussion

In this study, the detection rate of csPC was higher in the transperineal software fusion biopsy group than in the transrectal cognitive fusion biopsy group (52.2% vs. 36.5%, p = 0.036). Through multivariate regression analysis, we observed that age, PI-RADS score > 3, and transperineal biopsy route were independent predictors of csPC. In addition, the transperineal biopsy group had an insignificantly lower rate of sepsis than the transrectal biopsy group (0% vs. 1.2%).

Pepe et al. first compared transrectal and transperineal software-fusion TB in the same patient [11]. The detection rates for csPC were 78.1% and 89.1% using the transrectal and transperineal approaches, respectively. Notably, transrectal TB missed 53.3% of cancers in the anterior zone, whereas transperineal TB missed 13.3% of cancers in the anterior zone. Ber et al. also reported a superior detection rate of csPC using transperineal fusion compared with the transrectal fusion approach (42% vs. 26%) in a within-person study [20]. However, a meta-analysis of software fusion biopsy by Loy et al. showed similar sensitivity and specificity between the transperineal and transrectal approaches [14]. Another meta-analysis by Tu et al. enrolled patients undergoing software fusion, cognitive fusion, or in-bore targeting and suggested that transperineal biopsy was superior to the transrectal approach in detecting csPC (detection rate 62.2% vs. 41.3%, odds ratio 2.37, 95% CI 1.71–3.26) [13]. In line with previous studies, this study’s data showed that any prostate cancer detection rates were similar for both approaches (transperineal vs. transrectal, 60.9% vs. 56.5%, p = 0.659). However, transperineal biopsy detected more csPC than transrectal biopsy (52.2% vs. 36.5%, p = 0.036).

There are some explanations for the higher csPC detection rate with transperineal biopsy than that with transrectal biopsy. First, from an anatomical point of view, transperineal biopsy can easily assess the anterior zone or apex [21, 22]. Certain needle guides have been developed for sampling the anterior or apical prostate without tilting the ultrasound probe during transrectal biopsy; however, these equipment are not popular, and over-deflection of the needle would compromise tissue sampling [23]. In this study, the target lesions were located in the anterior zone in 39.4% and 33.6% of the transperineal and transrectal biopsy groups, respectively. We observed that the detection rate for csPC in the anterior zone by transperineal biopsy was insignificantly higher than that by transrectal biopsy (63.9% vs. 46.7%, p = 0.097). Second, in the lithotomy position, instead of the lateral decubitus position, the orientation of the prostate would be more similar to that in the supine position for MRI. Therefore, transperineal biopsy could reduce registration error compared with transrectal biopsy. Third, a linear ultrasound transducer was used for transperineal biopsy, whereas a convex transducer was used for transrectal biopsy. Compared with the convex transducer, the linear transducer might cause less prostate deformation and increase registration accuracy. Finally, the transrectal biopsy cohort was collected several years before the transperineal biopsy cohort. Therefore, improvements in prostate mpMRI interpretation could impact csPC detection rates.

In this study, we demonstrated the safety of transperineal biopsy. This study showed that 18.5% of men undergoing transperineal biopsy had AUR, which is comparable to the previous literature [24–26]. This higher rate of AUR compared with transrectal biopsy might be explained by the increased number of total biopsy cores. Furthermore, it is noteworthy that none of the men who underwent transperineal biopsy developed sepsis, whereas 1.2% of men who underwent transrectal biopsy had sepsis. Sepsis is a non-negligible risk following transrectal biopsy, despite bowel preparation and antibiotic prophylaxis. Although transperineal biopsy is more time-consuming and expensive, it can significantly reduce infectious complications compared with transrectal biopsy [27]. Therefore, the latest EAU guidelines recommend that the transperineal route be considered first for prostate biopsy [5]. The small sample size used in this study may explain why there was no significant difference in the sepsis rate between the transperineal and transrectal biopsies. Hence, a larger-scale study is needed to assess infectious issues between transperineal and transrectal fusion biopsies.

In this study, we used different methodologies for lesion targeting in these two cohorts, including software fusion in transperineal biopsy and cognitive fusion in transrectal biopsy, due to the development timeline of prostate TB at our institute. In 2012, we began performing prostate TB without software assistance. After the introduction of the MR/US fusion platform, we performed MR/US software fusion biopsy exclusively. Cognitive registration is an important basis for MR/US software fusion biopsies [28, 29]. In addition, cognitive registration is a reasonable alternative for lesion targeting [30]. This study data showed no significant difference in the detection rate for csPC by TB alone between software and cognitive fusion biopsies (43.5% vs. 29.4%, p = 0.094). Current literature also suggests comparable cancer detection rates between software and cognitive fusion biopsies [7, 8]. However, in this study, both TB and SB were performed by the same urologist who was not blinded to MRI, which may explain the higher csPC detection by transperineal SB. Moreover, the data of this study provided a real-world experience, which could enrich comparative studies of prostate MRI-TB.

It is noteworthy that both transperineal and transrectal prostate biopsies are feasible under local anaesthesia. A growing body of literature shows that transperineal biopsy under local anaesthesia could be a safe, tolerable, and effective method, with acceptable cancer detection rates [31, 32]. However, most patients in this study were anxious about the procedure, and none wanted to have any painful experiences. Therefore, all transperineal fusion biopsies in our series were performed under general or spinal anaesthesia. With improved biopsy techniques, transperineal biopsy under local anaesthesia may be our goal in the future.

This study has some limitations. First, it was not a randomised controlled study, and the number of cases was limited. However, the study cohorts were prospectively collected and compared; therefore, selection bias could be minimised. Second, patients with PI-RADS 1 or 2 lesions on MRI were not enrolled, precluding us from comparing positive and negative imaging results. Third, a combination of TB and SB was used as the reference standard instead of radical prostatectomy. Notably, the mean total biopsy cores were up to 20 and 26 in the transrectal and transperineal biopsies, respectively. The increased number of biopsy cores may help decrease the false-negative rates of biopsies [33]. Finally, a single fusion platform was used in this study; therefore, the results might not be generalisable to patients undergoing TB using other fusion platforms.

## Conclusion

In this comparative study, transperineal MR/US software fusion biopsy detected more csPC than transrectal cognitive fusion biopsy in patients with MRI-visible prostate lesions. In addition, transperineal biopsy carried minimal risk for infectious complications. These results must be validated in large-scale studies.

## Supplementary Information

**Additional file 1.** Checklist for START criteria.

## Data Availability

All data generated or analysed during this study are included in this published article.

## Abbreviations

TRUS: Transrectal ultrasound
SB: Systematic biopsy
MR/US: Magnetic resonance/ultrasound
csPC: Clinically significant prostate cancer
ciPC: Clinically insignificant prostate cancer
mpMRI: Multi-parametric magnetic resonance imaging
TB: Targeted biopsy
EAU: European Association of Urology
AUA: American Urological Association
DRE: Digital rectal examination
PSA: Serum prostate-specific antigen
PI-RADS: Prostate Imaging-Reporting and Data System
START: Standards of Reporting for MRI-Targeted Biopsy Studies
T2WI: T2 weighted imaging
DWI: Diffusion-weighted imaging
IQR: Interquartile range
AUR: Acute urinary retention
