# Supplementary material for: A comparative study of transperineal software-assisted magnetic resonance/ultrasound fusion biopsy and transrectal cognitive fusion biopsy of the prostate
Source: BMC Urol. 2022 Apr 29;22:72. doi: 10.1186/s12894-022-01011-w (PMC9052657; doi:10.1186/s12894-022-01011-w)
Supplement: Supplementary file 1 — Additional file 1. Checklist for START criteria. [file 12894_2022_1011_MOESM1_ESM.docx]

| V | 1. Identification as a study reporting results from MRI-targeted biopsy of the prostate, with a clear statement of the study aim |
| --- | --- |
| V | 1. The study design (eg, prospective or retrospective, cohort or randomized) |
| V | 1. The dates of recruitment, including whether any men have been included in previously published cohorts. |
| V | 1. Whether recruitment was based on PSA values alone or results from other tests, such as MRI, TRUS, or biopsy. |
|  | 1. The biopsy and treatment status of the population, specifying: |
| V | 1. Number of men with prior biopsy negative for cancer |
| V | 1. Number of men with prior biopsy positive for cancer and the number of men in each Gleason score category (eg, 3 + 3, 3 + 4, 4 + 3, 4 + 4) |
| V | 1. Number of men with previous treatment to the prostate |
| V | 1. Summary measures (range and mean or median) for age, prostate volume, and prebiopsy PSA |
| V | 1. A flow chart of the number of men who were suitable for study inclusion, those who were then excluded (with reasons specified), and those who completed the study |
|  | 1. The field strength of the magnet, specific coils used (eg, pelvic, endorectal), and a brief description of the sequences, including: |
| V | 1. Slice thickness and true acquisition resolution based on the field of view and reconstruction matrix for T2-weighted imaging, DWI, and DCE |
| V | 1. For T2-weighted imaging, which planes were acquired |
| V | 1. For DCE, the temporal resolution and the model used for postprocessing |
| V | 1. For DWI, the b-values used, which image sets were analysed (eg, high b-value image, ADC map, or both) and whether qualitative or quantitative analysis was carried out |
| V | 1. The reporting method used, including the use of any scoring system for suspicion of prostate cancer, whether a prose or diagrammatic report was used, and whether the radiologist was blinded to the clinical information |
| V | 1. The experience of the reporting radiologist (eg, number of years) |
| V | 1. Whether targeted cores or standard cores were taken first, whether they were potted separately, and the approach used for each technique (eg, transrectal, transperineal) |
|  | 1. The method of registration and guidance used for MRI-targeted biopsy, including: |
| V | 1. The type of registration used (eg, visual or software registration) |
| V | 1. For visual registration, whether the biopsy operator had direct access to the MRI images or used a prose or diagrammatic report |
| V | 1. For software registration, the software name and version, the MRI-sequence used for registration, and whether registration was rigid or non-rigid |
| V | 1. The guidance used during the biopsy procedure (eg, ultrasound or MRI) |
| V | 1. Whether the person taking the standard cores was aware of the location of the lesion on MRI |
| V | 1. The number of men who had an MRI with a suspicious lesion and the number who had an MRI-targeted biopsy |
| V | 1. A summary measure (mean or median) of the number of targeted cores taken per prostate or per lesion and of the number of standard cores taken per prostate |
| V | 1. The number of men in each Gleason score category (eg, 3 + 3, 3 + 4, 4 + 3, 4 + 4) from targeted cores alone and standard cores alone |
| V | 1. The number of men with clinically significant and clinically insignificant cancer detected by standard cores alone and targeted cores alone, with the criteria used for the definition of clinically significance |
|  | 1. A cross-tabulation of the number of men with clinically significant and clinically insignificant cancer detected by targeted biopsies against the number detected by standard biopsies |
|  | 1. The proportion of cores positive for clinically significant cancer in targeted cores alone and standard cores alone and the mean number of cores taken per diagnosis of clinically significant cancer for each technique |
|  | 1. A comparison between targeted and standard biopsy techniques for: |
|  | 1. Proportion of cores positive for clinically significant cancer |
|  | 1. Sampling efficiency (eg, mean number of cores taken per diagnosis of clinically significant cancer) |
| V | 1. Number of men diagnosed with clinically significant and clinically insignificant cancer |

Supplementary table: Checklist for START criteria
